# Pathogenesis, Clinical Considerations, and Treatments: A Narrative Review on Leprosy

**DOI:** 10.7759/cureus.49954

**Published:** 2023-12-05

**Authors:** Phat H Le, Sandy Philippeaux, Tiombee Mccollins, Cassande Besong, Alexander Kellar, Vincent G Klapper, Adalyn S De Witt, Joseph Drinkard, Shahab Ahmadzadeh, Sahar Shekoohi, Giustino Varrassi, Alan D Kaye

**Affiliations:** 1 School of Medicine, American University of the Caribbean, Miramar, USA; 2 School of Medicine, Ross University, Miramar, USA; 3 Department of Internal Medicine, Louisiana State University Health Sciences Center, Shreveport, USA; 4 School of Medicine, Indiana School of Medicine, Indianapolis, USA; 5 Department of Anesthesiology, Louisiana State University Health Sciences Center, Shreveport, USA; 6 Pain Medicine, Paolo Procacci Foundation, Rome, ITA

**Keywords:** immunologic reactions, armadillo, mycobacterium leprae, blindness, leprosy

## Abstract

Hansen disease, known as Leprosy, is an infectious disease caused by *Mycobacterium leprae*. The disease was once thought to be highly contiguous, and patients with leprosy were treated poorly and had to face discrimination due to the gruesome disease’s complications. *Mycobacterium leprae*, the bacterium causative of leprosy, can generally be found in the nine-banded armadillo. The bacterium is transmitted via aerosol droplets and broken skin-to-skin contact. Once M*. leprae* enters the body, it will target peripheral nerves and the lining mucosa of the skin and eyes, thus causing inflammation and tenderness of the affected area. Over time, this will lead to peripheral neuropathy and weakness of the affected body parts. Treatment of leprosy involves multi-drug combinations such as dapsone, rifampin, and clofazimine. Even though leprosy is curable, early detection and treatment are crucial to preventing irreversible damage and disabilities. Prevention measures include early detection, treatment regimen adherence, close contact prophylaxis, contact tracing, and community awareness. This review aims to provide the latest diagnostic and therapeutic recommendations for leprosy. It outlines the epidemiology, microbiology, clinical treatment, and immunological methods used to detect leprosy.

## Introduction and background

Leprosy is a contagious infection that is caused by *Mycobacterium leprae*. The disease causes damage to the affected area by targeting the peripheral nerves, which results in swelling of the affected area. The infection commonly targets the nerves, eyes, skin, and mucosal lining. Thus, the affected area will lose the ability to be sensitive to pain and touch, putting the patient at risk for injuries such as cuts and burns, which can lead to infection [1-5].

*M. leprae *is a pathogen that has adapted to a specific environment. *Mycobacterium leprae* is an intracellular organism that targets nerves and results in the clinical symptoms of leprosy. It is weakly acid-fast and has undergone significant genome reduction, leaving it with the smallest genome among mycobacteria and many non-functional pseudogenes. Related to the non-functional pseudogenes, it is challenging to culture the organism in a laboratory [1,4,6,7]. Through its evolution, *M. Leprae *has learned how to evade the host's immune system, thus increasing its chance of survival. Using the phenolic glycolipid I (PGL-1), a surface lipid of *M. Leprae*'s cell wall,* M. Leprae *can defend itself against oxidative killing. Plus, *M. leprae* can survive and multiply within macrophages, allowing it to escape the host immune system; thus,* M. Leprae *can stay dormant inside the host system for a long time until the symptoms appear. *M. Leprae* prefers cooler temperatures and is typically found in lower-temperature areas of the skin. Its viability decreases rapidly above 35°C, and because of this, most animals cannot be infected with *M. Leprae* as they clear the bacteria quickly. The only animal reliably developing leprosy with neurological involvement similar to humans is the nine-banded armadillo, the only natural host of *M. Leprae* other than humans. Once *M. Leprae* infects an area, the skin area will typically change color, becoming lighter or darker, often becoming dry and flaky. The affected area can lose feeling or even become red due to inflammation [1,8,9]. Leprosy is a significant global health concern, but despite the stigma, it is not as highly contagious as commonly believed. Effective treatments are available, but early diagnosis is crucial to prevent irreversible disability in the eyes, hands, and feet due to neuropathy. Lifelong care may be necessary for these disabilities. This article reviews leprosy's epidemiology, microbiology, clinical manifestations, diagnosis, and discussions regarding issues related to treatment.

## Review

Methods

This is a narrative review. The sources for this review are as follows: searching on PubMed, Google Scholar, Medline, and ScienceDirect using keywords: Leprosy, Blindness, Mycobacterium leprae, Armadillo, and Immunologic reactions. Sources were accessed between August 2023 and November 2023.

Epidemiology

Over the decades, Mycobacterium leprosy has been feared as a highly transmissible, life-debilitating disease. Current literature tells us it is difficult to spread and can be treated easily. Yearly incidence rates, per data reported globally to the World Health Organization in 2019, propose that approximately 150 people in the United States and 250,000 worldwide contract Leprosy. Children comprise 15,000 diagnosed cases within this significant number [1,10-14].

In the United States, 150 to 250 cases are reported yearly. Most occur in those who live in regions where the disease is still common. States reporting the highest number of new cases are Arkansas, California, Florida, Hawaii, Louisiana, New York, and Texas. Countries reporting the highest number of new cases are Brazil, Indonesia, and India. India alone produces over half of all new cases, highlighting the disproportionate transmission. Globally, 2 to 3 million individuals are living with Leprosy-related disabilities. Early diagnosis and treatment can prevent morbid complications, allowing those who develop leprosy to live a fully active life [10-14].

Transmission of Leprosy has yet to be entirely understood. Data shows evidence of human, wildlife, and environmental reservoirs that offer transmission pathways for *Mycobacterium leprae* and *Mycobacterium lepromatosis*. Individuals residing in close contact with infected leprosy patients were most likely infected via infectious aerosols. Aerosol droplets containing the bacteria are created by sneezing, coughing, and possibly through broken skin-to-skin contact. Once bacteria travel and infect the upper respiratory tract, extensive dissemination within the host can occur [1,15].

In the southern United States, the nine-banded armadillo (Dasypus Novemcinctus) is a proven natural host and reservoir of *M. leprae*. Identical strains (SNP subtype 3I-2-v15) are found to be passed zoonotically from armadillos to humans when they hunt, handle, or eat these animals. Nevertheless, most people encountering armadillos have a shallow risk of getting the disease. In 2016, *M. leprae*and *M. lepromatosis* were found in red squirrels (Sciurus vulgaris) in the United Kingdom. The isolated strain from red squirrels closely relates to the southern United States armadillo strain. Potentially, animal reservoirs relate to environmental exposure by shedding viable* M. leprae* bacteria. This could explain the stable global occurrence of leprosy. Multidrug treatment only lowers human-to-human transmission [15-19].

In experimental trial settings, *M. leprae* has been shown to survive inside amebae and amoebic cysts for weeks. This allows the amoebae to function as a vector in transmission. Typically, most people do not develop Hansen's disease following exposure. Various risk factors have been correlated with a higher chance of acquiring leprosy. Leprosy is endemic in regions within countries of Asia, Africa, and North and South America. Being in close contact with an individual with untreated leprosy increases one's chances of contracting the disease compared to the general population. Various studies have proposed that contacts of patients with the pervasive lepromatous leprosy strain have a higher risk than those with the limited tuberculoid leprosy strain. In the southern US, the nine-banded armadillo has been found to be naturally infected and transmit leprosy. Immunosuppression in cases such as HIV, organ transplantation, and chemotherapy increases predisposition to this disease [10-14,19].

Genetic variation determining how the human body responds to infection has also been suggested to impact one’s chance of contracting leprosy. The immunologic reaction to leprosy is determined by innate and adaptive immunity. Variations in genes of the NOD2-mediated signaling pathway can impair the innate immune response. Clinical manifestations can vastly vary depending upon the body’s ability to mount an acquired immune response to the bacteria infection. This cellular immune response appears to be regulated by numerous non-human leukocyte antigen (HLA) genes [10-14,19].

Microbiology

*Mycobacterium Leprae* has an affinity for infecting the skin and nerves of the body. Leprosy is a nonfatal contagious disease that is the most common cause of non-traumatic peripheral neuropathy worldwide [1,8]. *Mycobacterium Leprae* (*M. leprae*) is an intracellular acid-fast bacillus, aerobic and rod-shaped, that can infect humans and other species such as armadillos and primates. It can be transmitted and exit via the skin, specifically the dermis and nasal mucosa. *Mycobacterium leprae* is not usually found in the epidermis of the skin. Still, according to multiple research, it has been found that there was evidence of *Mycobacterium leprae* being found in the desquamating epithelium of the skin and the superficial keratin layer of the skin, which strongly proves that this microorganism can survive and multiply alongside the sebaceous glands [1,4,5,8,10,20]. Although the exit route of *Mycobacterium leprae* is known and heavily researched, the entry route is still not defined and needs to be definitively debated about the upper respiratory tract and the skin; according to some recent research, the upper respiratory tract is most likely favored. Recently, some new studies revealed the entry route of *Mycobacterium leprae* is through the endoneurial laminin-2 isoform and the receptor alpha-dystroglycan; this newly found evidence will shed light on the pathogenesis of peripheral neural damage caused by Leprosy. Alpha dystroglycan is usually associated with the early development and pathogenesis of muscular dystrophy, but in the settings of *Mycobacterium leprae*, it serves as a receptor for Schwann cells [4,7,16,21].

Growth and incubation period

*Mycobacterium leprae* is an obligate intracellular pathogen that affects Schwann cells and macrophages, with a slow-growing time of 14 days. Mycobacteria differ from other bacteria because they have a unique lipid, mycolic acids, that makes up their membranes and gives them unique characteristics. This large hydrophobic cell membrane prevents polar molecules and most drugs from entering the cell [1-4,8,10,22]. The clinical manifestation of leprosy is highly dependent on the host immune response. Based on the host, they can develop either a T-cell-mediated immune response or a humoral-mediated immune response. Because *Mycobacterium leprae* does not respond to antibodies, patients developing a humoral-mediated immune response develop a much more severe clinical manifestation than those developing a T cell-mediated response. Patients whose immune system develops a T cell-mediated response develop antigen-specific CD4+ T-cells of the Th1 subtype. This has the same similarity as *Mycobacterium tuberculosis*. TH1 cells secrete cytokines such as IFN-gamma, activating macrophages and enabling them to phagocyte the bacteria. This pathway is described as “Tuberculoid Leprosy.”

Patients whose immune systems developed a humoral response produce antigen CD4+ T-cells of the TH2 subtype, which secretes cytokines such as IL-4 and IL-5. These interleukins stimulate antigen-specific B-cells. Because *M. Leprae* does not respond to antibodies, these individuals cannot fight this bacteria effectively and present with what is called “Lepromatous Leprosy” [1-4,8,10,22].

Clinical manifestations and diagnosis

The clinical manifestations of leprosy depend upon the body’s cell-mediated immune response. After several years of incubation, leprosy presents slowly with a various spectrum of disease. The most common subtypes are tuberculoid leprosy and lepromatous leprosy. A general presentation of fatigue and fever may exist within both types. The chief signs of leprosy include skin lesions, hypoesthesia, and peripheral neuropathy [1,10,16].

Tuberculoid leprosy is a milder form of the disease. It has a better prognosis in most patients but may progress to a more extreme form. Tuberculoid is characterized by painless red or pale lesions with loss of sensation on the face, trunk, and extremities. There is a palpable thickening of peripheral nerves because *M. Leprae* invades and multiplies within Schwann cells. The significant sensory loss makes patients vulnerable to trauma, infections, or muscle atrophy. With progression, lesions tend to obliterate the standard skin organs such as sweat glands and hair follicles. A vigorous cell-mediated immune response causes phagocytic destruction of the organism but also amplifies allergic reactions [2,5,10,16].

Lepromatous leprosy, a more severe form, involves widespread skin involvement with many bacteria. Thickening of peripheral nerves with paresthesia is present but lasts longer than seen in tuberculoid leprosy patients. Repeated trauma of the hands and feet allows superinfection to occur with potential ulceration. Through disease progression, facial deformities and paralysis develop. The typical leonine facial appearance results from the loss of eyebrows and eyelashes thickening and enlargement of the nostrils, ears, facial skin, and cheeks. In the late stages of advanced disease, the gradual destruction of the nasal septum causes it to collapse. Involvement of bones, eyes, and other tissues can ensue. A weak cell-mediated immune response allows many organisms to remain viable in the lesions. Indeterminate leprosy, borderline tuberculoid leprosy, mid-borderline leprosy, and borderline lepromatous leprosy are intermediate forms of disease that may progress to either subtype [1,4,7,13,22-26].

Neuropathy is a common clinical symptom of leprosy. *M. leprae *infects peripheral nerves by invading lymphatic and epineural blood vessels. Upon reaching the endoneurium, it grows intracellularly within Schwann cells. *M. leprae* invades Schwann cells through a cascade of events. It begins by binding to α-dystroglycan, which is a component of the basal lamina. Once inside, it stimulates and attracts macrophages. Infected macrophages begin to produce nitric oxide that destroys axons by causing mitochondrial injury and the initiation of demyelination. Also, the complement system contributes to demyelination seen in patients, called rapid Wallerian degeneration. The bacteria may further promote the spread of infection by reprogramming Schwann cells to the progenitor stem cell stage. Tuberculoid leprosy is characterized by neuropathy of the face, trunk, and extremities. Nerve thickness is prominent and palpable because the bacteria multiply within the nerve sheaths. Impaired sensation exposes the patient to recurrent trauma and secondary infections [1,4,7,13,22-26]. Ophthalmic injury occurs in greater than 70% of leprosy patients. Blindness can arise in 5%. Impairment of ocular nerves that control muscles of the eyelids and provide sensory innervation to the cornea may lead to corneal ulceration or abrasion, drying of the cornea, and, most commonly, lagophthalmos. Each patient should be carefully evaluated for damage to the cornea, conjunctiva, and capability to close the eyelids fully. Early detection is vital for patient management [1,4,7,13,22-26]. There are two types of leprosy reactions: Type 1 and Type 2. These reactions appear to have different underlying immunologic mechanisms. The development of new lesions during or after treatment completion can usually be attributable to an immunologic response. Type 1 reaction (T1R, reversal reaction) occurs in patients with borderline tuberculoid, mid-borderline, or borderline lepromatous disease. Without treatment, the likely course of T1R is several months. T1R results from a spontaneous heightening of the cellular immune response and delayed-type hypersensitivity to *M. leprae* antigens. No known risk factors or routine laboratory tests are available to predict which patients may experience this reaction. Therefore, no changes should be made to a patient's treatment routine to avoid a reaction. Type 2 reaction (T2R, erythema nodosum leprosum, ENL) occurs in patients with borderline lepromatous and lepromatous disease. Without treatment, the likely course of T2R is one to two weeks, but it can reoccur over many months. The mechanism of T2R has yet to be fully understood. It is commonly deemed as an immune complex disorder. Risk factors for Type 2 reaction include pregnancy, lactation, and puberty [25,27-31].

Treatments 

Early clinical diagnosis and treatment are instrumental in reducing the transmission of leprosy and preventing the development of severe complications. Before pharmacological therapy, patients have undergone prednisolone challenge or skin biopsy with PCR testing to assess for known genetic markers of drug resistance. This allowed for a more effective treatment plan that ensured a lower probability of treatment failure. Due to the rising risk of bacterial resistance to therapy, like tuberculosis, the treatment options for leprosy consist of a multidrug approach, precisely, a three-drug regimen. According to the guidelines of the National Hansen’s Disease Program (NHDP), which is also supported by the World Health Organization (WHO), the first-line medications include Dapsone, Rifampin, and Clofazimine. Treatment alternatives (second line) for patients who failed a first-line anti-leprosy treatment or when drug resistance is detected include Ofloxacin and minocycline [5,11,13,23,26,32-40].

First-line triple therapy with dapsone, rifampin, and clofazimine are the most effective in the treatment of leprosy, but they do carry certain risks. Dapsone contains bacteriostatic activity that inhibits bacterial synthesis of dihydrofolic acid, thereby inhibiting bacterial nucleic acid synthesis and replication. Prior to the imitation of treatment, all patients should be screened for glucose-6-phosphate dehydrogenase deficiency, as dapsone may cause hemolytic anemia in these patients. Other adverse reaction of dapsone includes hypersensitivity syndrome, methemoglobinemia, and agranulocytosis. Moreover, rifampin contains bactericidal activity that inhibits bacterial DNA-dependent RNA polymerase, thereby preventing the elongation of the messenger RNA. The effect impedes RNA synthesis and results in cell death. Some notable drug side effects include Cytochrome P450 activation, hepatotoxicity, drug-induced hepatitis, and thrombocytopenia. In addition to the other agents, clofazimine contains bactericidal and anti-inflammatory activity that binds to mycobacterial DNA, thereby impeding bacterial growth. Some significant drug side effects include red-black skin discoloration, retinopathy, nephrotoxicity, and cardiac arrhythmia [5,11,13,23,26,32-40].

The second-line treatment is ofloxacin and minocycline for leprosy, which are fundamental to tackling the emerging problem of drug resistance within first-line drugs. Ofloxacin bacteriostatic activity inhibits bacterial topoisomerase IV and DNA gyrase, thereby preventing protein synthesis. Common side effects include headache, tendonitis, peripheral neuropathy, and hepatotoxicity. Minocycline is a fluoroquinolone that contains bactericidal activity that binds to bacterial 30S ribosomal subunit, thereby preventing protein synthesis [32]. Notable side effects include dizziness, photosensitivity, hypersensitivity reactions, and autoimmune disorders. Although drug resistance can occur with both drugs, they are less common and contain less severe adverse reactions than first-line agents [5,11,13,23,26,32-40]. The treatment course generally lasts about 12 months, daily or monthly; this depends on the clinical manifestation of the disease course as defined by the Ridley-Jopling classification. Leprosy disease presents clinically in a spectrum, reflecting the organism’s load and a patient’s immune response. The “Ridley-Jopling Classification” is a categorical division of leprosy that combines “the cutaneous, neurological, and biopsy findings, with the immunological capabilities” of the patients. The following are the classifications of leprosy: Tuberculoid (TT), Borderline tuberculoid (BT), Mid-borderline (BB), Borderline lepromatous (BL), Lepromatous (LL), and Indeterminate (I); nonetheless, “the majority of patients fall into a broad borderline category between TT and LL [5,11,13,23,26,32-40].

A significant complication associated with the treatment of leprosy is the possibility of relapse. Considering the prolonged treatment period, patient non-compliance and adherence to therapy are important risk factors, so patient education and follow-up assessment are essential. While these first and second-line drugs are generally efficient, complications and drug resistance can still arise, especially with dapsone and rifampin. Monitoring for these complications is crucial during the treatment course (Table 1) [5,11,13,23,26,32-40].

**Table 1:** The first and second-line medications for leprosy Information adapted from Leprosy: Treatment and prevention – UpToDate [1,32]

Drug [1,32]	Mechanism of Action	Adverse Reactions	Important Information
Dapsone	Bacteriostatic activity that inhibits bacterial synthesis of dihydrofolic acid, thereby inhibiting nucleic acid synthesis.	Hypersensitivity syndrome, methemoglobinemia, agranulocytosis and hemolytic anemia.	All patients should be screened for glucose-6-phosphate dehydrogenase G6PD deficiency prior to initiation therapy. Drug resistance is common.
Rifampin	Bactericidal activity that inhibits bacterial DNA-dependent RNA polymerase, thereby preventing the elongation of the messenger RNA.	Cytochrome P450 activation, hepatotoxicity, drug-induced hepatitis, and thrombocytopenia.	Drug resistance is common.
Clofazimine	Bactericidal and anti-inflammatory activity that binds to mycobacterial DNA, thereby inhibiting bacterial growth.	Skin discoloration, retinopathy, nephrotoxicity, and cardiac arrhythmia. `	Drug resistance is rare.
Ofloxacin	Bacteriostatic activity inhibits bacterial topoisomerase IV and DNA gyrase, thereby preventing protein synthesis.	Headache, tendonitis, peripheral neuropathy, and hepatotoxicity.	Drug resistance can occur.
Minocycline	Bactericidal activity that binds to bacterial 30S ribosomal subunit thereby preventing protein synthesis.	Dizziness, photosensitivity, hypersensitivity reactions, and autoimmune disorders.	Drug resistance can occur.

Prevention

A recent study examined the effectiveness of using rifapentine to prevent the spread of leprosy among household contacts of those diagnosed with the disease. The trial involved 7,450 individuals aged 10 years or older in China and found that a single dose of rifapentine significantly reduced the incidence of new leprosy cases over four years compared to no intervention. However, the reduction was not statistically significant compared to a single dose of rifampin. These results suggest that postexposure prophylaxis with rifapentine should be administered to household contacts of patients with newly diagnosed leprosy who are aged 10 years or older [32].

Prevention of leprosy is instrumental in reducing complications and transmission rates. Key preventive measures include early diagnosis and initiation of appropriate treatment, adherence to treatment regimens, prophylaxis for close contacts, contact tracing and surveillance, and health education and community awareness. These methods allow for early detection and treatment and encourage early reporting and treatment-seeking behavior. Implementation of preventive measures is paramount in controlling leprosy and reducing its burden on the communities [22,23,32,41-43].

## Conclusions

Leprosy is a contagious infection caused by *Mycobacterium leprae* and *Mycobacterium lepromatosis*. Leprosy is a nonfatal infectious disease that is the most common cause of non-traumatic peripheral neuropathy worldwide. It is estimated that 250,000 people contract leprosy every year. Areas with the highest transmission rates are Brazil, Indonesia, and India. However, international travel is so prevalent that the infection is not isolated to these areas. In the United States, 150 to 250 new cases are reported yearly. Transmission of the infection is not fully understood; however, the two proposed transmission routes are aerosol droplets and broken skin-to-skin contact. It is postulated that extensive dissemination within the host can occur once bacteria travel and infect the upper respiratory tract. The clinical manifestations of leprosy depend upon the body’s cell-mediated immune response. After several years of incubation, leprosy presents slowly with a various spectrum of disease. Tuberculoid and lepromatous forms of leprosy are the most prevalent subtypes. A common symptom of fatigue and fever may be present in both cases. Leprosy is characterized primarily by skin lesions, hypoesthesia, and peripheral neuropathy. Early physical exam findings include hypopigmented or reddish skin patches, diminished sensation in involved areas, paresthesia, painless wounds, and tender, enlarged peripheral nerves. The infection causes damage by targeting the peripheral nerves, which results in swelling of the affected area. The disease commonly targets the nerve eyes, skin, and mucosal lining. The loss of eyelashes and eyebrows and the thickening and enlarging of the nose, ears, facial skin, and cheeks contribute to the typical leonine facial appearance.

In summary, leprosy is a significant global health concern, but despite the stigma, it is not as highly contagious as commonly believed. Effective antimicrobial treatments are available for leprosy; however, due to the severe and lasting complications, early recognition and treatment are crucial to prevent irreversible disabilities of the eyes, hands, and feet. This literature review provides some knowledge on the epidemiology, microbiology, clinical manifestations, diagnosis, and treatment of leprosy, which can and should be used by healthcare professionals to diagnose, treat, and prevent the spread of disease and long-term disability.
